# Targeting ferroptosis in melanoma: cancer therapeutics

**DOI:** 10.1186/s12964-023-01296-w

**Published:** 2023-11-23

**Authors:** Khatereh Khorsandi, HomaSadat Esfahani, Saeedeh Keyvani- Ghamsari, Parisa lakhshehei

**Affiliations:** 1 Department of Photodynamics, Medical Laser Research Center, Yara Institute, ACECR, Tehran, Iran; 2 Department of Microbiology, Karaj Branch, Islamic Azad University, Karaj, Iran; 3 Department of Biochemistry, Faculty of Biological Sciences, North Tehran Branch, Islamic Azad University, Tehran, Iran

**Keywords:** Ferroptosis, Melanoma, Cell death, Cancer treatment, Targeted therapy

## Abstract

Melanoma is an aggressive kind of skin cancer; its rate has risen rapidly over the past few decades. Melanoma reports for only about 1% of skin cancers but leads to a high majority of skin cancer deaths. Thus, new useful therapeutic approaches are currently required, to state effective treatments to consistently enhance the overall survival rate of melanoma patients. Ferroptosis is a recently identified cell death process, which is different from autophagy, apoptosis, necrosis, and pyroptosis in terms of biochemistry, genetics, and morphology which plays an important role in cancer treatment. Ferroptosis happens mostly by accumulating iron and lipid peroxides in the cell. Recently, studies have revealed that ferroptosis has a key role in the tumor’s progression. Especially, inducing ferroptosis in cells can inhibit the tumor cells’ growth, leading to back warding tumorigenesis. Here, we outline the ferroptosis characteristics from its basic role in melanoma cancer and mention its possible applications in melanoma cancer treatment.

Video Abstract

**Supplementary Information:**

The online version contains supplementary material available at 10.1186/s12964-023-01296-w.

## Introduction

Melanoma is the most invasive skin cancer, and metastatic melanoma has the highest risk of death with a median survival rate of nearly 6 months [1]. Melanoma prevalence is significantly rising all around the world [2, 3]. Specialized pigment cells are known as melanocytes, which are found in the basal epidermis, and lead to melanoma [4]. In a normal physiological condition, keratinocytes control melanocyte growth and activity [4]. Due to abnormalities in critical genes that control cell growth, melanocytes cannot adequately respond to regulatory cues from keratinocytes, which ultimately results in aberrant growth. Melanoma can develop without a precursor lesion, although in certain instances, the development of a nevus or mole marks the beginning of this aberrant growth [5]. Melanoma has also been identified from transformed stem cells. Stem cell markers such as CD20, and CD133, as well as OCT 4, NANOG, and pSTAT 3, have been recognized in melanoma [6, 7]. A challenge in treating melanoma is the variety of cell populations with stem cell characteristics since some of these cells are resistant to therapy [8]. Cancer stem cells are also known to secrete factors in response to hypoxia, increasing tumor angiogenesis, and thereby promoting disease progression [9]. In spite of the development mechanism of melanoma, neovessel formation precedes tumor progression.

Current cancer treatment methods include surgical resection, chemotherapy, photodynamic therapy, immunotherapy, and targeted therapy. Depending on the patient's health, tumor stage, and location, the therapeutic strategy may consist of single drugs or combined therapies. Due to the development of different resistance mechanisms, the efficacy of various treatments may be decreased. Studies of the genetic profile of melanocytes and the discovery of molecular factors involved in the pathogenesis of malignant transformation have provided new therapeutic targets [10]. Today, two main new therapeutic strategies are routinely used which are molecularly targeted therapy (using dabrafenib, vemurafenib, encorafenib, trametinib, cobimetinib, binimetinib) and immunotherapy (using pembrolizumab, nivolumab, ipilimumab). Additionally, in the case of the presence of mutations in genes other than *BRAF* (B-Raf proto-oncogene, serine/threonine kinase), alternative targeted therapy may be considered, e.g., with imatinib, when a mutation in the *c-KIT* gene is present. It is also possible to treat injectable melanoma with the genetically modified oncolytic virus (talimogene laherparepvec) [11]. Adjuvant therapy with kinase inhibitors (dabrafenib and trametinib) and immunotherapy (pembrolizumab, nivolumab, ipilimumab) for high-risk melanoma are also registered [12] Although many patients take advantage of these new therapies, some patients do not respond to both targeted and immunological tratment. The development of reliable markers of response would allow for better personalization of the treatment and consequently would lead to improved patient survival and lower costs of patient care [13, 14]. For patients with solitary melanoma metastasis, metastasectomy is the standard of care, and chemotherapy may be recommended in some metastatic melanoma instances [15]. Radiotherapy can be effective for the treatment of skin, bone, and brain metastases, despite being rarely advised for original tumor treatment. It was claimed that the combination of photodynamic therapy (PDT) with chemotherapy (dacarbazine) is an effective treatment for reducing resistance in pigmented and unpigmented metastatic melanomas [16].

**Fig. 1:**
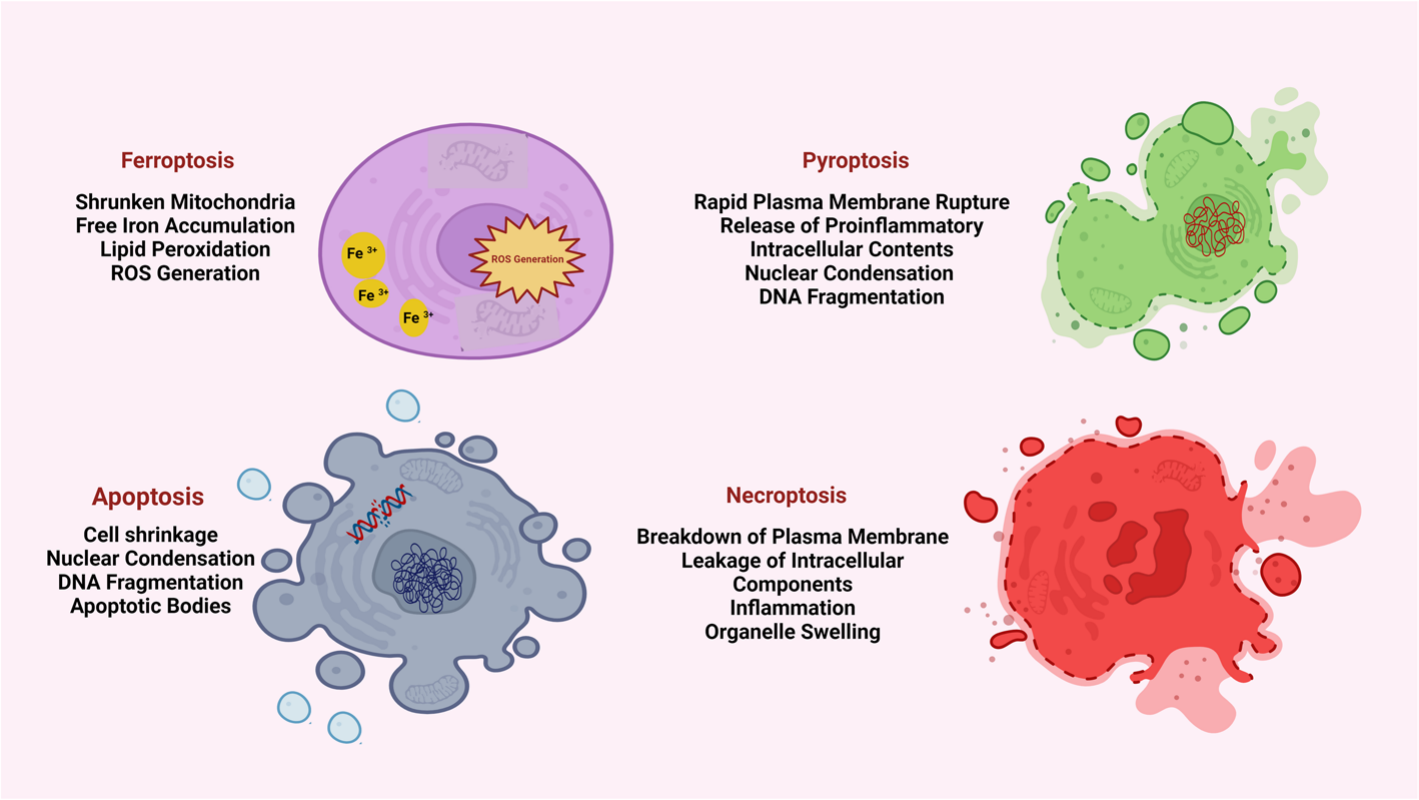
The main morphologic characteristics of cell death are apoptosis, pyroptosis, ferroptosis, and Necrosis. Apoptosis is characterized by DNA condensation and fragmentation, and the occurrence of apoptotic body. Cells with pyroptosis present DNA condensation and fragmentation, and the membrane is ruptured. Ferroptosis is defined as free iron accumulation, lipids peroxidation, ROS generation. Cells undergoing necrosis show DNA degradation and membrane rupture. Pyroptosis and necrosis are accompanied with cell membrane rupture and severe inflammatory reaction, while apoptosis and ferroptosis are devoid of these changes and there are no cell membrane alterations and no DNA fragmentation

Cell death entities can be categorized into programmed or non-programmed cell death based on their signal dependency. Programmed cell death (PCD) is driven by tightly regulated intracellular signal transduction pathways. By contrast, accidental cell death is referred to as non-PCD as a result of unexpected cell injury. Given the morphological characteristics and molecular mechanisms, PCD can be further categorized into apoptotic cell death and non-apoptotic cell death. Apoptosis retains cell membrane integrity and occurs in a caspase-dependent manner. By contrast, non-apoptotic cell death is mostly characterized by membrane rupture and caspase independency (Fig. 1).

Until ferroptosis identification as such in 2012, studies describing what is now known as ferroptotic cell death were attributed to alternative cell death mechanisms or not recognized as biologically significant [17]. In 2003, Dolma et al. identified erastin, a novel drug that had a selectively lethal effect on RAS-expressing cancer cells, although the mode of cell death was distinct from what had previously been observed. There were no nuclear morphological alterations, DNA fragmentation, or caspase activation, and caspase inhibitors had no effect on this process [18]. Subsequently, Yang [19] and Yagoda [20] found that This pattern of cell death is inhibited by iron chelating agents, whereas another substance, RSL3, can induce this pattern of cell death. The term ferroptosis was coined in 2012 to [21] describe an iron-dependent, non-apoptotic form of cell death triggered by erastin and RSL3. This discovery was followed by the development of ferrostatin-1, the first small molecule ferroptosis inhibitor, and the demonstration of glutamate-induced ferroptosis in organotypic rat brain slices, indicating the possible role of ferroptosis in neurodegeneration. Subsequently, numerous other researches began to demonstrate a similar ferroptotic process as the underlying mechanism for a variety of phenomena. In the presence of serum, amino acid starvation has been found to trigger non-apoptotic and non-necrotic cell death in multiple cell types [22, 23]. It was demonstrated that cystine deficiency was sufficient to cause the same serum-dependent cell death pathway and that transferrin, an iron carrier, was the essential serum component for cell death. Thus, cystine deficiency was similar to system Xc inhibition. These findings confirmed that ferroptosis is the cell death process triggered by amino acid starvation in the presence of an external iron source. Later, it was discovered that not all reactive oxygen species (ROS) function equally in ferroptosis, and that lipid peroxidation is the main factor of ferroptotic death [24], which has been supported by the identification of lipophilic antioxidants as inhibitors of ferroptotic death induced by erastin and other compounds [20].

In addition to revealing mechanisms of a fundamental biological process, cell death research has contributed to the development of novel cancer treatments over the past three decades. Knowledge of this mechanism has enabled the development of treatments that kill cancer cells by directly activating the cell death machinery and by synergizing with conventional chemotherapy and targeted therapies to improve cancer patients' outcomes [25]. The roles of autophagy-dependent cell death, necroptosis, ferroptosis, pyroptosis, and parthanatos have recently attracted considerable interest. This is despite the fact that melanoma cells are generally equipped with anti-apoptotic machinery and that recurrent genetic alterations in the RAS/RAF/MEK/ERK signaling significantly contribute to the pro-survival phenotype of melanoma [26]. In addition, the links between sensitivity to non-apoptotic cell death pathways and distinct cell morphologies have been identified, suggesting that the plasticity of melanoma cells can be exploited to alter their response to various cell death stimuli [26]. Increasing evidence indicates that the anti-tumor approach based on non-apoptotic cell death is a direction to solving existing problems in cancer treatment. On the one hand, numerous kinds of non-apoptotic cell death successfully bypass or overcome the resistance of tumor cells to apoptosis and provide alternative death pathways when the apoptosis pathway is deficient, so considerably enhancing the anti-cancer efficacy (Fig. 1) [27].

According to the studies ferroptosis can be introduced as a target for melanoma cancer treatment. In this review, we will describe ferroptosis and its mechanisms which are involved in different melanoma cancer therapies.

## Ferroptosis definition and its morphological hallmarks

In 2012, Dixon has defined the concept of ferroptosis as an iron-dependent form of cell death described by the excessive accumulation of lipid peroxides and reactive oxygen species (ROS) [21]. Recent data has shown that ferroptosis has a role in the occurrence and progression of a variety of diseases, making it the central issue of controversy in current research about to the treatment and prognosis improvement of related disorders. Electron microscopy shows that morphologically, Ferroptosis is characterized by decreased mitochondrial volume, increased bilayer membrane density, and decreased or disappearance of mitochondrial cristae. However, there is no evidence of the cell membrane permeabilize, nucleus fragments, or chromatin condensation. Although oxidative damage in the DNA occurs by some activators of ferroptosis, the nucleus shows a normal size, without chromatin condensation [28]. In some cases, other features like detachment and rounding up of cells and an increased autophagosome is observed in ferroptosis cells [21, 29, 30]. In addition, ferroptosis is a defined form of inflammatory regulated cell death (RCD) that immune cell infiltration that could be observed in tissues with ferroptotic damage. For instance, acute pancreatitis is an inflammatory disorder characterized by an initial injury that results in acinar cell death. Ferroptotic acinar death contributes to experimental pancreatitis in mice, particularly when circadian rhythms are disrupted [31]. According to hematoxylin and eosin stain, the histological assessment revealed ferroptosis is associated with leukocyte infiltration and pancreatic damage [31].

## Biochemical hallmarks of ferroptosis

Ferroptosis is a ROS-dependent form of controlled cell death characterized by two primary biochemical characteristics, iron accumulation and enhanced lipid peroxidation. Ferroptosis is mainly triggered by intracellular glutathione (GSH) depletion and a decrease in the activity of glutathione peroxidase 4 (GPX4). As a result, lipid peroxides are unable to be metabolized by the GPX4-catalyzed reduction mechanism, which results in an accumulation of lipid peroxides. Fe^2+^ triggers ferroptosis by oxidizing lipids in a Fenton-like way and by producing a large amount of reactive oxygen species [30].

### Iron overload

Multiple iron metabolism regulators are involved in the process of ferroptosis. As they enhance intracellular iron accumulation, the common ferroptosis activators erastin and RSL3 block the antioxidant system. Iron can mediate the production of excessive ROS via the Fenton reaction and contribute to increased oxidative damage [21]. Heme and non-heme iron in excess can directly induce ferroptosis [32]. Arachidonate lipoxygenase (ALOX) and EGLN (also known as PHD) 2-oxoglutarate (2OG)-dependent dioxygenases, which are in control of lipid peroxidation and oxygen homeostasis, are two iron-containing enzymes that activity may be increased by iron. The sensitivity of ferroptosis is influenced by local and systemic cellular iron regulation [33]. Iron overload- or the usage of iron-chelating agents-related suppressor genes may successfully prevent ferroptosis cell death. It is not well understood why only iron (no other metals like zinc) also induces ROS generation via a Fenton reaction [34] to trigger ferroptosis [21]. This may be happening due to the iron overload and activate specific downstream effectors that participate in the performance of ferroptosis after the generation of lipid ROS.

### Lipid peroxidation

Lipid peroxidation happens under conditions where ROS readily react with vulnerable lipids on cell membranes. Initial lipid hydroperoxides (LOOHs) and later reactive aldehydes, such as malondialdehyde (MDA) and 4-hydroxynonenal (4HNE), which rise during ferroptosis, are examples of lipid peroxidation's products. Saturated fatty acids (no double bond), monounsaturated fatty acids (MUFAs, 1 double bond), and polyunsaturated fatty acids (PUFAs, > 1 double bond) are the three different types of fatty acids. Only the peroxidation of PUFAs in phospholipids by ALOXs appears to be necessary for ferroptosis among all the cell membrane lipids that can be oxidized, including phosphatidylcholine, phosphatidylethanolamine (PE), and cardiolipin [35, 36]. Although there are extensive ultrastructural changes in mitochondria during ferroptosis, there is no evidence of cardiolipin peroxidation (Fig. 1) [37].

### Genetic features

Genetically, multiple genes have been found to regulate ferroptosis [19, 20]. Ferroptosis particularly involves genetic changes in iron homeostasis and lipid peroxidation metabolism. However further study is needed to determine the specific regulatory mechanisms. Prostaglandin-endoperoxide synthase 2 (PTGS2/COX2), a crucial enzyme in prostaglandin production, is an example of how overexpression of a select few genes/proteins has been considered to be a genetic signature of ferroptosis [38]. The up-regulation of PTGS2 mRNA is used as a pharmacodynamic marker of ferroptotic tissues in mice exposed to erastin or RSL3 [38]. Although it is a widely used biomarker of ferroptosis in vitro or in vivo, PTGS2 inhibitor (e.g., indomethacin) fails to affect ferroptotic cell death indicating it is not a contributor of ferroptosis. In contradiction, MIR212-mediated downregulation of PTGS2 mRNA prevents ferroptotic neuronal death in a traumatic brain injury mouse model [39] suggesting a cell type-dependent role of PTGS2 in ferroptosis. Further mechanism studies suggest that the up-regulation of PTGS2 gene expression in ferroptosis requires lipid peroxidation because antioxidant vitamin E or toxic 4-HNE can inhibit or induce PTGS2 expression in cancer cells or macrophages, respectively [38].

**Fig. 2:**
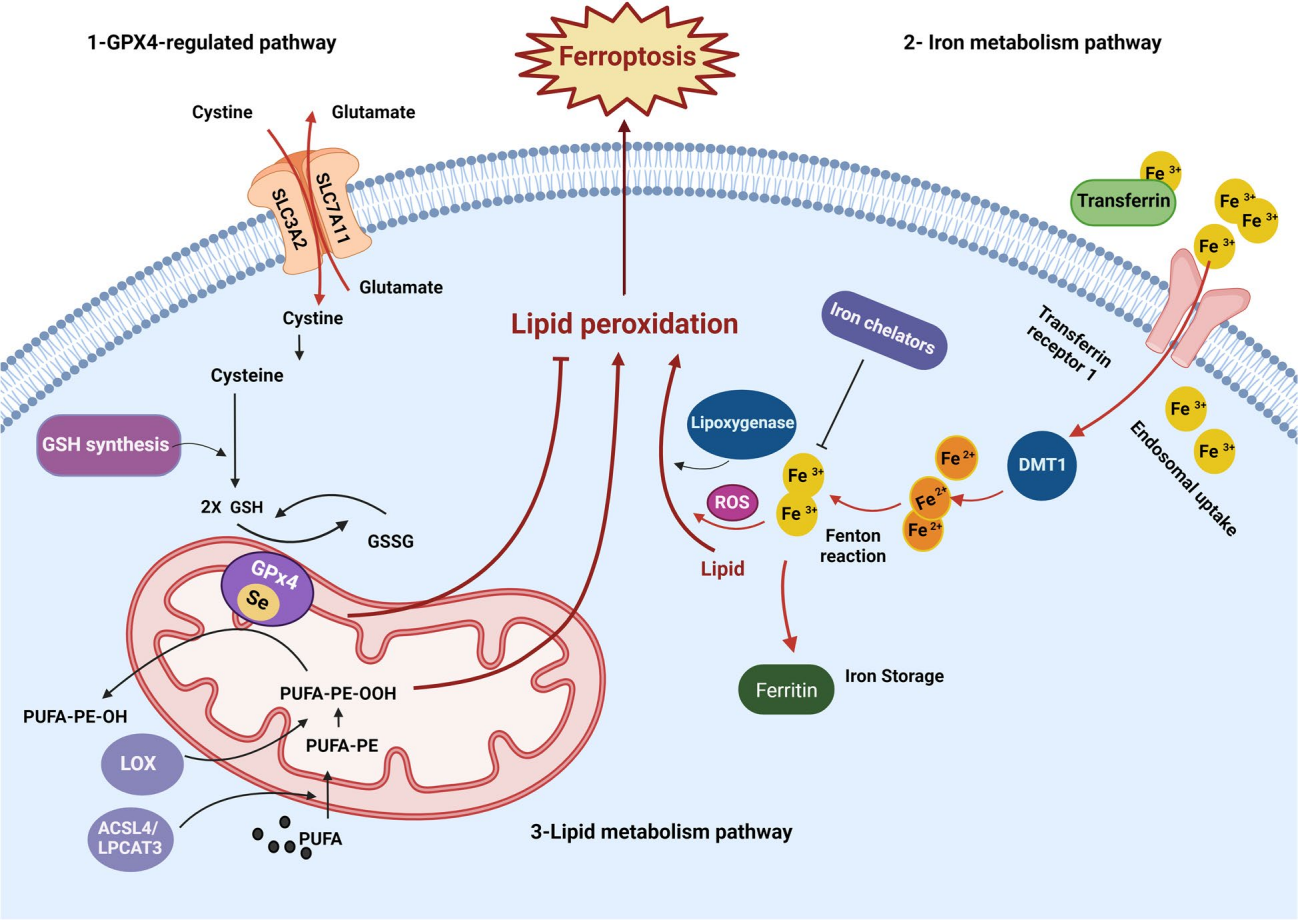
Ferroptosis cell death process. Ferroptosis is triggered by direct suppression of system Xc- or GPX4, which ultimately results in cell death. The ferroptosis process involves lipid ROS. On the one hand, PUFA peroxidation is thought to be a key element. In contrast, the execution of ferroptosis is based on iron overload

A specific biomarker for ferroptosis is the enzyme acyl-CoA synthetase long-chain family member 4 (ACSL4), which is involved in fatty acid metabolism. When ACSL4 is overexpressed, it increases the quantity of polyunsaturated fatty acids (PUFAs) in phospholipids, which are sensitive to oxidation processes and cause ferroptosis [35, 40, 41]. Nevertheless, ferroptosis is not dependent on ACSL4 in all circumstances. Specific conditions can cause a cell to undergo ferroptosis when ACSL4 is low (Fig. 2) [42]. Therefore, in response to ferroptosis signals, cells "decide" whether to live or die based on the balance of injury and anti-injury conditions.

## Selective ferroptosis inducers

Four classes of ferroptosis inducers can be classified:

### Class1: inhibit system Xc- and prevent cystine import

Erastin is one of the ferroptosis inducers which is identified by antioxidant depletion generated by cystine glutamate antiporter inhibition (xCT). Another xCT inhibitor ferroptosis inducer is the clinical medicine sulfasalazine, used to treat inflammatory bowel disease [17]. Cysteine is the rate-limiting substrate for the important antioxidant glutathione, when system XC − is inhibited results in a reduction of cysteine, as a substrate for GSH synthesis, which will result in diminished levels of GSH [38, 43]. For GPX4 to catalyze the degradation of hydrogen peroxide and hydroperoxide and prevent the production of L-ROS, GSH is a crucial cofactor. As a result, erastin indirectly reduces the synthesis of GPX4 and further reduces the potential of cells to produce antioxidants by inhibiting the system XC [44]. It has recently been shown that GPX4 activity was decreased in a number of cancer cells treated with erastin. These drugs induce ferroptosis, which has an anticancer effect [21, 22]. Nonetheless, erastin has another physiological target, voltage-dependent anion channels (VDACs), which cause mitochondrial dysfunction. Additionally, it was recently shown that erastin's activation of ferroptosis is related to an increase in the lysosomal-associated membrane protein 2a, which in turn generates chaperone-mediated autophagy and, subsequently, increases the destruction of GPX4 [45].

A particular inhibitor of the xCT-mediated cystine transporter is sulfasalazine (SAS) [46, 47]. As an anti-inflammatory drug, SAS has the ability to scavenge ROS, reduce the production of IL-1 and IL-2, and inhibit nuclear factor κ B (NFκB), as well as leukocyte motility [48–50]. SAS has also been identified as a GSH depletion inducer (90%). It can arrest tumor growth, and improve sensitivity to chemotherapeutic agents in pancreatic, prostate, and mammary cancer [51–53]. SAS prevents a cystine-glutamate transporter and therefore plays important role in the induction of ferroptosis [21, 54]. The iron-dependent lethal accumulation of lipid ROS can make this process more sensitive when the cancer cells display high levels of Ras activity or p53 [21, 54]. Both ferroptosis and glutathione depletion can be driven by xCT inhibitors such sulfasalazine, glutamine, and sorafenib [55].

### Class 2: inhibit GPX4:

RSL3 and DPI7, which directly inhibit GPX4 activity and induce ferroptosis, are classified as the second category. GPX4 is a key modulator of ferroptosis by inhibiting the formation of lipid peroxides. The enzyme GPX4 reduces the cytotoxic lipid peroxides (L-OOH) to the corresponding alcohols and converts GSH into glutathione disulfide (GSSG) (L-OH). GPX4 inhibition stops the conversion of lipid peroxides to lipid alcohols, which leads to the accumulation of lipid peroxides, which is a hallmark of Ferroptosis. RSL3, a ferroptosis inducer, directly interacts with GPX4 and suppresses its activity, reducing cells' capacity to defend against ROS and causing ferroptosis [38]. Additionally, the DPI7 and DPI10 compounds directly affect GPX4 and cause ferroptosis.

### Class 3: degrade GPX4, bind to SQS and deplete antioxidant CoQ10

A member of the third category is FIN56, which has two ways to induce ferroptosis. First, FIN56 stimulates the degrading of GPX4. Second, FIN56 binds to the squalene synthase enzyme, which causes the endogenous antioxidant coenzyme Q10 to be depleted (COQ10). This procedure makes cells more sensitive to ferroptosis caused by FIN56 [56]. GPX4 protein levels are negatively regulated by FIN56, which also activates squalene synthase (SQS), a mevalonate pathway enzyme that functions downstream of HMG-CoA reductase and leads to ferroptosis. In one mechanism, ACC activity is required for FIN56 to enhance the degradation of GPX4 in a process. Tofa's inhibition of ACC prevents GPX4 from being degraded by FIN56, however, the link between FIN56, ACC, and GPX4 degradation is unclear. The second pathway involves the binding and activation of SQS, an enzyme that converts farnesyl pyrophosphate (FPP) into squalene. This decreases the amount of FPP available for protein prenylation and metabolite synthesis, which inevitably leads to the depletion of coenzyme Q10 (CoQ10). SQS inhibition increases the pool of FPP and its derivative products that are accessible, decreasing ferroptosis [57].

### Class 4: oxidizing ferrous iron and directly inactivating GPX4 through lipidome

The final category includes FINO2 exerting ferroptosis by the dual-function effect of oxidation of labile iron and the inactivation of GPX4 [56]. The initiation of ferroptosis by FINO2 is highly dependent on the availability of iron and oxidizes a wide range of polyunsaturated lipids. FINO2 is able to initiate ferroptosis preferentially over other types of cell death, in contrast to other peroxide-containing compounds. Numerous elements can be considered for this remarkable selectivity, including the inactivation of GPX4, or the lipophilicity of FINO2. FINO2 can accumulate in lipid bilayers of cell membranes according to its high lipophilicity, causing oxidized PUFAs directly and triggering ferroptosis in the locations [58].

## Ferroptosis-inducing nanoparticles in cancer

In recent years, researchers have tried to combine bio-nanotechnology with ferroptosis to develop candidates with a stronger antitumor effect [59, 60].

The delivery of nano-drugs is based on engineering technology. Nanoparticles are used to deliver and control drug release and adjust the intracellular chemical reaction to affect the ROS levels, thereby improving the pharmacokinetic properties of the drug [61, 62]. Nanomaterials can be used to supplement exogenous lipids in tumor cells to increase the accumulation of intracellular lipid peroxides, promote ferroptosis, and achieve the goal of curing cancer [63]. The current emerging nano therapies mainly focus on inhibiting the expression of GPX4 in tumor cells, increasing the accumulation of ferrous/iron ions in tumor cells, and regulating lipid peroxidation [64–66].

This process primarily involves triggering or promoting the Fenton response in tumor cells [64].

Some nanomaterials, such as sorafenib, are encapsulated into network-like nanostructures composed of Fe^3+^ and tannic acid (TA) [67]. Sorafenib is a typical small-molecule System Xc- inhibitor. It inhibits GPX4, leading to tumor-specific ferroptosis, and TA is used to chemically reduce Fe^3+^ to Fe^2+^ and continuously supply Fe^2+^ to maintain the iron redox cycle and maintain the Fenton reaction [67]. Shen et al. by using lactoferrin (LF) and RGD dimer (RGD2)-coupled cisplatin (CDDP) Fe_3_O_4_/Gd_2_O_3_ hybrid nanoparticles FeGd-HN@Pt@LF/RGD2 successfully combined and delivered Fe^2+^, Fe^3+^, and H_2_O_2_ (the reactants involved in the Fenton reaction) to the tumor sites. Their local concentration was increased to accelerate the Fenton reaction, significantly improving the efficacy of in situ brain tumor ferroptosis treatment [68]. In another study, Professor Song Yang and Associate Professor Zhu Xiaokang from Southwest University designed a poly-nanosystem Fe3O4-PLGA-Ce6 coated with PLGA, containing iron oxide (Fe_3_O_4_) and photosensitizer Ce6, and used it to synergize ferroptosis–photodynamics anticancer treatment. Fe_3_O_4_-PLGA-Ce6 nanosystem can dissociate in an acidic tumor microenvironment and release ferrous/iron ions and Ce6. Subsequently, the released ferrous/iron ions will react with excess hydrogen peroxide in the cell to produce a Fenton-like reaction generating hydroxyl free radicals (•OH), and induce ferroptosis of tumor cells [69].

Novel nanoparticles were reported as important in inhibiting tumor progression as presented in Table 1 [67, 70, 71].

**Table 1:** The applications of nanomaterials to target tumor ferroptosis

Nanomaterials	Target	Mechanisms of action	References
SRF@FeIIITA	GPX4	Inhibit GPX4 enzyme for ferroptosis initiation	[67]
AMSNs	GSH, GPX4	Highly efficient glutathione (GSH) depletion ability	[70]
FeGd-HN@Pt@LF/RGD2	GPX4	Accelerate Fenton reaction and generates ROS to induce ferroptosis	[68]
SPFeN	GPX4	Generates hydroxyl radicals and accelerates the Fenton reaction	[72]
Fe_3_O_4_-PLGA-Ce6	GSH, GPX4, SLC7A11	Accelerate Fenton reaction and generates ROS to induce ferroptosis	
LDL-DHA	GPX4	Experience pronounced lipid peroxidation, depletion of glutathione, and inactivation of GPX4	[73]

## Ferroptosis's role in melanoma

One of the most aggressive and challenging treatments among human cancers is skin melanoma whose annual incidence is rising. Over 60% of all fatal skin malignancies are cutaneous melanomas, the most dangerous form of skin cancer that results from melanocyte transformation. Melanoma has a substantial socioeconomic impact due to its high mortality rate in the metastatic form and its disproportionately high incidence in young adults [74]. It is significant to highlight that nevi, benign collections of melanocytes, are produced when melanocytes grow unevenly, but dysplastic nevus is thought to be a possible precursor to cutaneous melanoma since it displays a high level of cytologic and architectural atypia [75]. When tumor cells do not exhibit a significant proliferation capacity or metastasis, they are in the radial growth phase (RGP), which is the first observable malignant stage. Tumor cells can infiltrate the dermis as an increasing mass during the vertical growth phase (VGP), the main lesion, and subsequently move into the lymphatic and blood arteries, causing systemic dissemination. The progression to the invasive stage is accelerated by the accumulation of the initial genetic alterations that occur during the precursor stage. The final stage of tumor development is metastasis (metastatic melanoma) [76].

Various factors have been considered to involve in melanoma progression [4], namely genetic alteration in multiple genes (oncogenic and tumor suppressor genes) such as cyclin-dependent kinase inhibitor 2A (CDKN2A), melanocortin receptor (MC1R), cyclin-dependent kinase 4 (CDK4), Ras, and BRAF (v-raf murine sarcoma viral oncogene homolog B1) genes. A list of onco-suppressor and oncogenic factors involved in melanoma is presented in Table 2.

**Table 2:** Onco-suppressor and oncogenic factors involved in melanoma

Gene	Gene type	Function	Comment	References
*MC1R*	Oncogenic	The eumelanin pigments (dark brown pigments) are synthesized in response to UV exposure by this receptor	The high expression leads to the more frequent cell division	[77]
*CDK4*	Oncogenic	Contributing to the regulation of the cell cycle	Triggering metastasis-inducing pathways and also, interfering the phosphorylation of pRB (retinoblastoma protein) in the mid-G1 phase	[78]
*BRAF*	Oncogenic	Contributing to regulating cell division and differentiation as a part of the family of signal transduction protein kinases	Activating the MAPK pathway involved besides RAF and the RAS family	[79]
*CCND1*	Oncogenic	In a manner dependent on cyclin-dependent kinases, or CDKs, promote progression of G1-S phase of the cell cycle by inactivating the RB protein	Contributing to the phosphorylation of pRB by binding to CDK4	[80]
RAS and NRAS (neuroblastoma RAS viral oncogene homolog)	Oncogenic	Regulating cell division by encoding N-Ras protein as GDP–GTP-regulated binary on–off switches	Activation of MAPK and the phosphatidylinositol 3-kinase (PI3K) pathway	[81]
*c-KIT*	Oncogene	Interacting with stem cell factor (SCF), activating downstream signaling molecules, causing the expression of certain genes, regulating cell differentiation and proliferation, and restraining cell apoptosis, associated with tumor formation, development, migration, and recurrence	Induction of both MAPK and PI3K-AKT kinase pathways	[82]
*GNAQ* (guanine nucleotide-binding protein G(q)) and *GNA11* (guanine nucleotide-binding protein subunit α-11)	oncogene	Making a guanine nucleotide-binding protein G(q) subunit alpha (Gαq) to activate downstream cellular signaling pathways	Encoding G-protein alpha subunit q and alpha subunit 11, respectively	[83]
*P53*	tumor suppressor gene	controlling cell division and cell death in the cell’s nucleus	Associated with advanced-stage disease	[84]
*TP 53*	tumor suppressor gene	Encoding P53 protein as a tumor suppressor by keeping cells from growing and dividing	A somatic mutation causing abnormal p53 expression	[85]
*P16*	tumor suppressor gene	As a CDK inhibitor; it slows down the progression of the cell cycle	Effecting G1 cyclin-dependent kinases cell regulator	[86]
*BCORL1*	tumor suppressor gene	Encode a transcriptional corepressor binding to promotor regions of DNA binding proteins	Represseing E-cadherin expression via interaction with CtBP	[87]
*PPP2R3B* (gonosomal protein phosphatase 2 regulatory subunit B, beta)	tumor suppressor gene	As a major family of Ser/Thr phosphatase gene negatively control cells growth and division	Intervening with DNA replication and cell cycle progression by its regulatory subunit PR70	[88]
*RASA2 (RAS p21 protein activator 2)*	tumor suppressor gene	Encode RasGAP as a tumor suppressor	Activation of RAS GTPase, increase RAS activation, and melanoma cell growth	[89]
*PTEN*	tumor suppressor genes	Regulate cell division by keeping cells from growing and dividing	Elimination of negative regulating on downstream components of the PI3 kinase pathway and Akt	[90]
*CDKN2A*	tumor suppressor genes	Encode the cell cycle inhibitor P16^CDKN2A^	Disruption of the function of p16INK4a and p14ARF effecting two cell cycle regulatory pathways, the p53 and the RB1 pathways	[91]

Indeed, despite significant advancements in the therapeutic management of human cancers in recent years, patients with metastatic melanoma still have not greatly benefited from these medical developments. To establish and define successful treatments to consistently improve the overall survival rate of patients affected by this malignancy, new worthwhile therapeutic techniques are urgently required [92]. Recently, it has also been demonstrated that ferroptosis is related to resistance to cancer therapy. Additionally, a number of studies have suggested that controlling ferroptosis may affect the effectiveness of cancer treatment and perhaps overcome resistance [93–95]. Here, we give a thorough explanation of the mechanics behind ferroptosis and discuss how controlling it can treat melanoma cancer. Ferroptosis can initiate glutamate-induced cytotoxicity. Therefore, iron chelators and other ferroptosis inhibitors can suppress glutamate-induced cytotoxicity. Ferroptosis can also be regulated by glutaminolysis and glutamine metabolism in various ways.

For instance, glutamine is taken in and converted into glutamate and -ketoglutarate (-KG) by the glutamate importer (SLC1A5/SLC28A1), glutaminase (GLS), and glutamic-oxaloacetic transaminase-1 (GOT1). Inactivation of any of these genes may cause resistance of cells to ferroptosis [23]. Reduced SLC1A5 expression has been linked to increased ferroptosis, decreased glutamine synthesis, and decreased glutamine accumulation in melanoma [96]. Additionally, the reduction in glutamic-oxaloacetic transaminase prevented the depletion of Glu, consequently leads anti-ferroptosis action on melanoma cells [97].

According to Sato et al. study on melanoma pathogenesis and metastasis, ferroptosis initiate by inducing cysteine-glutamate antiporter (System X_c_^−^) deficient B16F10 melanoma cells. Deficiency in System X_c_^−^ resulting in a reduction of cysteine uptake, cellular glutathione, cell cycle progression, and proliferation in vitro, tumor spheroid formation ex vivo, and subcutaneous tumor formation in vivo. Notably, the ferroptosis inhibitor liproxstatin-1 was unable to reverse any of these alterations. Additionally, by using the tail vein, intrasplenic, IP, and footpad injections, loss of System Xc- generally have fewer metastases in vivo and is attached poorly to the lung vascular endothelium in vitro as well as reduced migration. The summary of the study isassessing the link between ferroptosis susceptibility and metastatic potential in melanoma [98]. Melanoma that metastasizes through the blood rather than the lymphatic system became dependent on the ferroptosis inhibitor GPX4. Cells with chemical ferroptosis inhibitors treatment metastases than were those that did not treat after intravenous, but not intra-lymphatic, injection. In this study, they observed differences between lymph fluid and blood plasma that may involve in the reduction of oxidative stress and ferroptosis in lymph, such as higher levels of glutathione and oleic acid and less free iron in the lymph. Oleic acid improved the ability of melanoma cells to generate metastatic tumors and prevented ferroptosis in an Acsl3-dependent manner. Melanoma cells in lymph nodes have shown resistance to ferroptosis. When intravenous injection was followed by metastases, these cells were more dominant than melanoma cells from subcutaneous tumors. Melanoma cells protected from ferroptosis which increase their capacity for survival during following metastasis through the blood [99].

## Ferroptosis in melanoma cancer-associated signaling pathways

Ferroptosis as programmed cell death is very important in the development and progression of cancer. Cell susceptibility to ferroptosis has been observed at different stages of melanoma progression. Ferroptosis was initially thought to occur only in RAS-mutant cancer cells, but it was later found that induction of ferroptosis could be independent of the mutated state of the RAS [100]. Given that the BRAF-activating mutations have been identified as the most common genetic variation in melanoma, BRAF inhibitors can increase the susceptibility of melanoma cells to ferroptosis. In fact, BRAF inhibition can activate an oxidative phosphorylation system in cells, induce ROS generation, and by altering the metabolism in the cell can increase ferroptosis [101–103]. It has been suggested that DNA damage can initiate ferroptosis in melanoma cells as well as several oncogenic pathways have been identified in melanoma, which predisposes cells to ferroptosis by affecting essential cell regulators [104]. Various regulators of ferroptosis have been identified in melanoma. TP53, which encodes P53, is mutated in many cancer cells. But its mutation in melanoma has been found to be very rare [105]. It is suggested that P53 function regulates ferroptosis by regulating cellular redox and metabolism. Researchers have observed that P53 suppresses SLC7A11 activity. Downregulation of SLC7A11 has been proposed as a marker of induction of ferroptosis in melanoma metastatic cells [100, 103]. It has been reported that inhibiting SLC7A11 activity, increases the efficacy of ferroptosis-promoting drugs in melanoma cells [92]. P53 also reduces the uptake of cysteine and acts as a rheostat in the cell due to the stimuli present. In the case of low oxidative stress, P53 reduces ferroptosis, while in the case of high ROS content, it increases ferroptosis. In fact, the expression of several ferroptosis-regulating proteins and redox homeostasis is regulated by P53 [100].

Iron metabolism also plays a key role in inducing ferroptosis in cancer cells, including melanoma. YAO et al., Showed that iron regulatory protein 1(IRP1) induced ferroptosis in melanoma cell lines A375 and G-361. It was observed that the expression of IRP1 and IRP2 were upregulated in the melanoma cells through the inducer of ferropotosis such as erastin and RSL3. IRP1 played a major role in regulating iron homeostasis and thus promoted ferroptosis, and IRP2 increased function IRP1. IRP1 regulated the expression of proteins involved in iron metabolism, such as transferrin receptor, ferroportin, and ferritin heavy chain 1 which increased ferroptosis by increasing the amount of intracellular iron [106].

High nuclear factor erythroid 2-related factor 2 (Nrf2) has also been observed in malignant melanoma cells, leading to the intrinsic resistance of cells to anticancer therapies [107]. Gagliardi et al. investigated the role of Nrf2 in ferroptosis-resistant melanoma cells. Their studies showed that Nrf2 expression was increased in ferroptosis-resistant cells that lead to the expression of glutathione-specific gamma-glutamylcyclotransferase 1 (CHAC1) and the aldo–keto reductase (AKRs). Expression of these markers reduced 12/15-LOX-generated lipid peroxides and inhibited ferroptosis. Inhibition of Nrf2 activity re-induced ferroptosis in the cells [103]. Zhu et al., also showed that Nrf2 regulated the expression of UV-induced programmed cell death ligand 1 (PD-L1) in melanoma cells. Targeted therapy of Nrf2 induced tumor infiltration via CD8 + and CD4 + T cells and inhibited tumor progression. They showed simultaneous inhibition of Nrf2 and anti-programmed cell death protein-1 (a checkpoint protein on T cells) increased melanoma cell death [108]. AKRs such as AKR1C1, AKR1C2, and AKR1C3 play an important role in ferroptosis cell death in melanoma cells. It has been observed that the activity of these genes inhibits the cell death of ferroptosis in melanoma cells by reducing the amount of lipid peroxide. Inhibition of AKRs led to lipid ROS production and the induction of ferroptosis in resistant melanoma cells [92].

MicroRNAs (miRNAs) also play an important role in regulating ferroptosis in melanoma. It has been observed that they regulate the process of ferroptosis in cells by regulating glutamate metabolism. For example, overexpression of miR-137 in melanoma cells downregulated the expression of glutamine transporter SLC1A5, reduced the process of lipid peroxidation, and the accumulation of iron, which reduced ferroptosis. Thus up-regulation of the miR-137 gene increased tumor growth, tumor volume, and drug resistance in melanoma cells [96]. Overexpression of miR-9, also down- regulated the expression of glutamic-oxaloacetic transaminase 1 (GOT1) in melanoma, decreased lipid peroxidation, and iron accumulation, causing cells to escape from ferroptosis. Conversely, inhibition of miR-9 function increased the susceptibility of melanoma cells to inducers of ferroptosis [97].

## Ferroptosis in melanoma cancer therapy

The research showed that certain subtypes of melanoma cells could be successfully treated using multiple therapies including chemotherapy, radiotherapy, and immunotherapy in combination with ferroptosis-inducing drugs [109]. Mechanisms of several ferroptosis inducers and their combination therapy in melanoma are listed in Table 3.

**Table 3:** Mechanisms of several ferroptosis inducers

Ferroptosis inducers	combination	Mechanism	References
Sorafenib	Vemurafenib	The combination therapy induced ferroptosis by reducing GSH concentration, increasing the production of ROS, MDA (an end product of lipid peroxidation), and iron	[110]
Fluvastatin	-	Downregulated the expression of GPX4	[111]
Vemurafenib	Trametinib	down regulated the expression of SLC7A11	[112]
Vemurafenib	Erastin or RSL3	Increased ferroptosis in resistance melanoma cells by targeting GPX4 and System Xc^−^ transporte	[113]
Dioscin	Rapamycin Cisplatin Dacarbazine Vemurafenib	ROS generation, upregulation of transferrin, downregulation of ferroportin Its combination with other drugs had synergistic effect	[114]
Radiotherapy	Immunotherapy	Reduced SLC7A11, and promoted lipid oxidation	[115]
FINs	Radiotherapy	Reduced SLC7A11 expression, inhibited GPX4 activity	[116]
Cyst(e)inase	Radiotherapy	Decreased GSH levels, The combination therapy enhanced lipid oxidation and had a synergistic effect on the melanoma cells	[117]
Gallic acid	Pre-irradiation	produced ROS, Reduced GPX activity and induced lipid peroxidation	[118]
Sulfasalazine	Radiotherapy	reduced repair of damaged DNA, and GSH concentration, and synergistically increased the effect of radiotherapy in the melanoma cells	[119]
Immune checkpoint blockade: anti-PD-L1, and anti-CTLA4	-	IFNγ secretion, xCT suppression, lipid ROS production	[120]
Radiation therapy	Cyst(e)inase, Anti-CTLA4, Anti- PD-L1	IFN release, xCT suppression, ATM activation, and lipid peroxidation	[121]
Buthionine sulfoximine	-	Inhibited the synthesis of GSH and induced lipid ROS	[94]
Fluvastatin	-	Decreased expression of GPX4	[94]
TGF-β inhibitors and PD-1 Antibodies	FINs	Increased the amount of H2O2, promoted the Fenton reaction, generated hydroxyl radicals	[122]
BAY-87–2243	vemurafenib	increased cellular ROS levels, stimulated lipid peroxidation, and reduced glutathione levels upregulate mitochondrial oxygen consumption and decrease glycolysis	[102]
ML162	-	GPX4 inhibition	[123]
ML210		GPX4 inhibition	[124]
RSL3	Lorlatinib	GPX4 inhibition ALK inhibitor	[125]
Erastin	oncolytic vaccinia virus (Immunotherapy)	System Xc inhibition	[126]
ICG001	Immunotherapy	Wnt inhibitor	[127]
Iridium (III) complex Ir-pbt-Bpa + ferrostatin-1	PDT and Immunotherapy	iron-dependent oxidative stress and/or glutamate toxicity	[128]

*FINs* Ferroptosis inducers, *ATM* Ataxia-telangiectasia mutated

## Chemotherapy

Despite the spread of chemotherapy drugs, their function has been limited due to drug resistance. Therefore, identifying new treatment goals seems necessary. In the meantime, ferroptosis has been considered by researchers as a new type of programmed cell death. Various studies have shown the importance of ferroptosis in the treatment of cancer cells. Also, the combination of chemotherapy and ferroptosis inducers has shown a significant synergistic effect on cancer cells [44, 94, 129]. On the other hand, dysregulation and ineffective ferroptosis lead to resistance of cancer cells to chemotherapy. Many chemotherapeutic drugs have been shown to induce ferroptosis in cancer cells by pharmacologically regulating or genetic pathways and eliminate the treatment resistance by targeting lipid metabolism, iron metabolism, and canonical GPX4-regulated pathways (Fig. 1) [130]. Tang et al., showed sorafenib enhanced the function of vemurafenib in vemurafenib-resistant melanoma A375 and SK-Mel-28 cells by inducing ferroptosis. The combination of sorafenib and vemurafenib reduced the concentration of GSH and increased the production of ROS, MDA (an end product of lipid peroxidation), and iron which led to ferroptosis [110]. Viswanathan et al. Reported that fluvastatin downregulated the expression of GPX4 in various cancer cells, including melanoma, thereby promoting ferroptosis [111]. Osrodek et al., showed that Vemurafenib and trametinib downregulated the expression of SLC7A11 in melanoma cells [112]. Vemurafenib, along with erastin or RSL3, also increased ferroptosis in resistance melanoma cells by targeting GPX4 and System Xc^−^ transporter [113]. The researchers showed that dioscin induced ferroptosis in melanoma cells by producing ROS and regulating the expression of transferrin and ferroportin, which caused an increase in intracellular iron. Dioscin in combination with chemotherapy drugs such as cisplatin, vemurafenib, rapamycin, and dacrbazine, also had synergistic effects in the melanoma cells [114]. Zeng et al., have shown that paclitaxel, nelarabine, dolastatin 10, actinomycin D, eribulin mesylate, vinorelbine, vinblastine, chelerythrine, docetaxel, and homoharringtonine are closely linked to ferroptosis in melanoma cells, the activation of ferroptosis showed good results in the patient survival. Therefore, they suggested that these drugs could be used as supplements or in combination with other drugs in the treatment of melanoma [131]. Generally, researchers showed the inducers of ferroptosis increased the therapeutic effects of chemotherapy in the melanoma cells (Fig. 2).

## Radiotherapy

Radiotherapy often causes cell death by causing breaks in DNA structure. It has also been shown that radiotherapy indirectly reduces GSH and increases ROS production by inducing cell water radiolysis and increasing oxidase activity (Fig. 2). The effectiveness of radiotherapy increases with the reduction of GSH [109]. Lang et al., reported the radiotherapy-induced ferroptosis in cancer cells, such as melanoma. They also showed that immunotherapy synergistically increased the sensitivity of tumors to radiotherapy by reducing SLC7A11 and inducing ferroptosis (Fig. 2) [115]. Another study reported ferroptosis inducers (FINs) such as sorafenib, RSL3, sulfasalazine, and erastin, synergistically increased the effect of radiotherapy in various cancers, including melanoma, by reducing SLC7A11 expression or inhibiting GPX4 [116]. The therapeutic effects of cyst(e)inase, a recombinant human enzyme, which causes the breakdown of extracellular cysteine and cystine, have been studied in tumor cells. Cyst(e)inase increased ROS production and cell death by decreasing intracellular GSH levels. Researchers reported cyst(e)inase combined with radiotherapy enhanced lipid oxidation and had a synergistic effect on B16F10 melanoma cells [117]. Khorsandi et al., reported that pre-irradiation increased the anti-cancer function of gallic acid in melanoma cells by producing ROS, reducing GPX activity, and inducing lipid peroxidation [118]. Nagane et al., reported sulfasalazine, an inhibitor of the cystine-glutamate antiporter, reduced repair of damaged DNA, and intratumorally GSH concentration in B16F10 melanoma cells and synergistically increased the effect of radiotherapy in the cells [119].

## Photodynamic therapy

Photodynamic therapy (PDT), a non-invasive and highly selective cancer treatment modality, has been studied in melanoma treatment. It involves the use of a photosensitizer activated by light to generate ROS, leading to localized cytotoxicity in tumor cells [132]. However, the efficacy of PDT in advanced melanoma still faces challenges that need to be addressed. It is worth exploring whether combining ferroptosis-targeted strategies with PDT can overcome the limitations.

The photodynamic treatment (PDT) and ferroptosis combined therapy were successful by loading methylene blue (MB) into SFT through the deposition of tannic acid (TA) and Fe3 + onto SRF nanocrystal [38]. Ferroptosis-induced nanomaterials can also happen through GSH metabolism. Based on the high surface area to volume ratio, the arginine-capped manganese silicate nanobubbles (AMSNs) were created with a high efficiency of GSH depletion [56]. According to an in-vivo investigation, AMSNs could suppress the formation of Huh7 xenograft tumors by downregulating GPX4. Liproxstatin-1, a ferroptosis inhibitor, might prevent this [57]. Researchers have synthesized a potent mitochondria-localized photosensitizer called cyclometalated Ir(III) complexes Ir-pbt-Bpa, which exhibits a strong antitumor impact on melanoma cells by inducing ferroptosis and restraining tumor growth in murine models [133].

Another study constructed a nanoparticle-based material named protoporphyrin IX-based polysilsesquioxane platform (PpIX-PSilQ NPs), which synergizes with PDT to mainly induce ferroptotic cell death by upregulating lipid peroxides and inactivation of GpX enzymes [134].

**Fig. 3:**
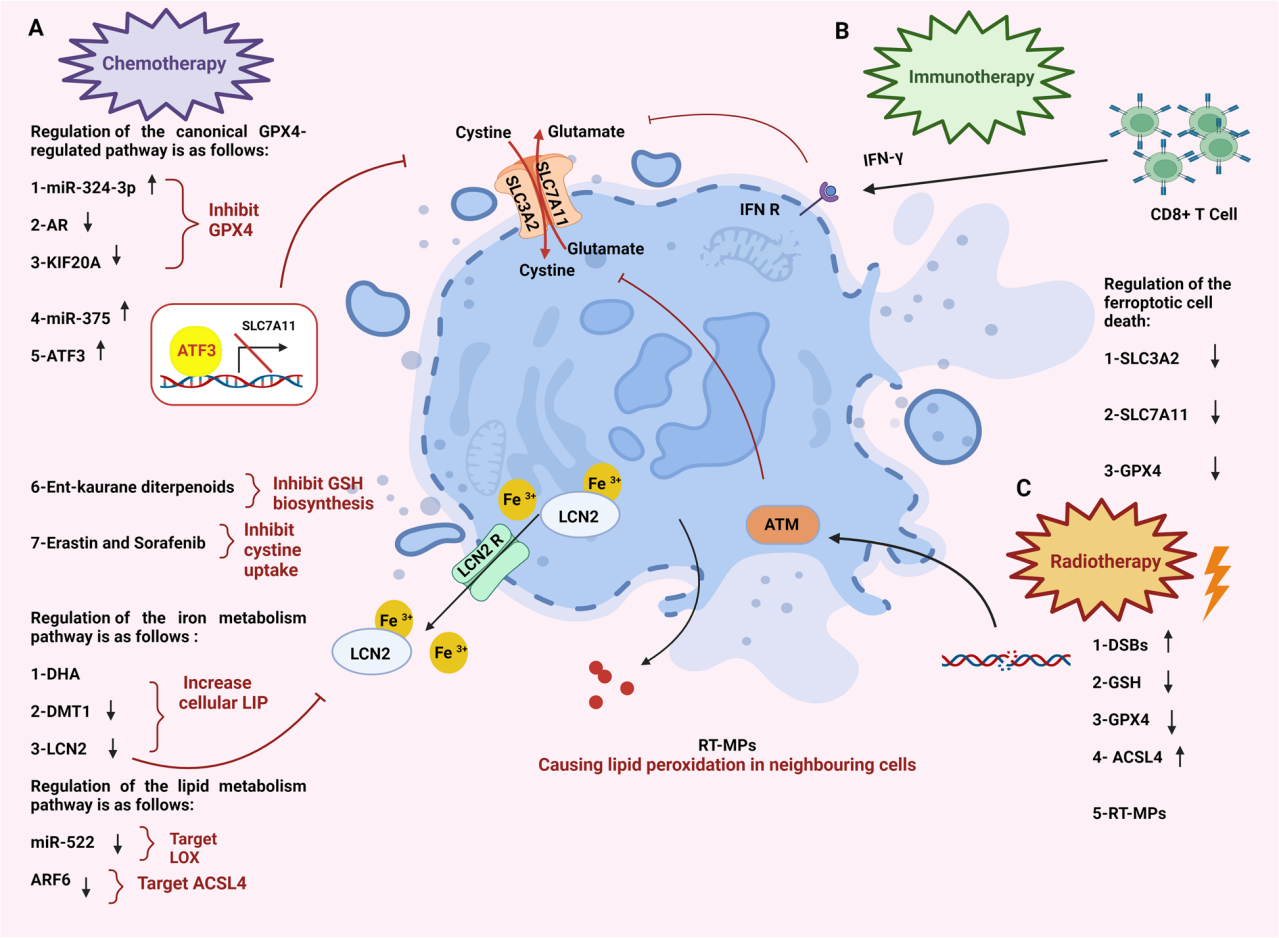
Mechanisms governing ferroptosis by radiotherapy, chemotherapy, and immunotherapy. A The canonical GPX4-regulated pathway, the iron metabolism pathway, and the lipid metabolism pathway are the three pathways that start the process of ferroptosis and chemotherapy resistance reversal. The canonical GPX4-regulated pathway is regulated as follows: Directly inhibit GPX4 via increasing miR-324-3p, decreasing AR and KIF20A, inhibiting GSH production with ent-kaurane diterpenoids, and blocking cystine absorption with erastin and sorafenib, miR-375, and ATF3. The iron metabolism pathway is regulated as follows: DHA increases cellular LIP while repressing DMT1 and LCN2. The lipid metabolism pathway is regulated as follows: Target LOX by decreasing miR-522 and ACSL4 by decreasing ARF6. B T cells that have been stimulated by immunotherapy treatments release interferon (IFN), which causes ferroptosis. IFN- may reduce tumor cells' ability to take up cystine, which reduces the effectiveness of intracellular GPX4. C There were four phases in the mechanism of radiotherapy-induced ferroptosis. First, radiotherapy impairs system XC transport via ATM, which in turn impacts GSH production. Second step: By increasing ACSL4 expression, radiotherapy encourages lipid production. Third step: By generating DNA damage, radiotherapy triggers autophagy-dependent ferroptosis. Fourth step: Radiation therapy makes it easier for RT-MPs to be made, which leads to lipid peroxidation in nearby cells

Hence, the use of combined ferroptosis-targeted strategies may provide alternative approaches in designing PDT to improve treatment outcomes Fig. 3.

## Immunotherapy

Today, despite many advances in the treatment of melanoma, most patients experience resistance mechanisms of treatment and patient survival is limited due to progression, invasion, and metastasis. Immunotherapy using immune checkpoint inhibitors has dramatically improved the treatment of melanoma the deadliest type of skin cancer [135]. Immunotherapy is a relatively new method of cancer treatment that helps the immune system removes cancer cells and despite its specific benefits and performance, creating resistance to it is a major treatment challenge. Mechanisms of resistance to immunotherapy consist of two parts: 1- Tumor cell-intrinsic factors and 2- tumor cell-extrinsic factors [136]. Intrinsic factors are related to changes in the tumor cells themselves, such as the up-regulation or down-regulation of specific genes and pathways that prevent the penetration or function of immune cells in the microenvironment of the tumor. Tumor cell-extrinsic factors include factors separate from the tumor cells in the tumor microenvironment, such as regulatory T cells, and inhibitory immune checkpoints, which lead to the inhibition of immunity against tumor cells and the development of primary and/or adaptive resistance [136]. Inhibition of immune checkpoints by activating CD + 8 T cells induced ferroptosis in tumor cells, including melanoma. The researchers reported overexpression of TYRO3-suppressed ferroptosis and increased resistance to α-PD-1/PD-L1 immune checkpoint inhibitors. Upregulation of TYRO3 has been suggested as one of the pathways of ferroptosis resistance in tumors. It was observed that up-regulation of TYRo3 was associated with lower survival of treated melanoma patients with α-PD- 1checkpoint inhibitors. In cells with TYRO3 overexpression, the expression of ferroptosis-inhibiting genes such as *SLC40A1, SLC7A11, SLC3A2,* and *GPX4* increased, while the genes that promoted ferroptosis such as *SLC5A1* and *TFRC* decreased (Fig. 2) [135].

Another study has shown that cytokines secreted by T cells such as TNF-α and IFNγ in melanoma cell culture medium induced dedifferentiation and increased ferroptosis in the cells through activating of NF-κB or STAT1 signaling pathways [113]. It has also been shown that immunotherapy by activating CD8 + cells increase lipid peroxidation in melanoma cells and activates ferroptosis as a cytotoxic pathway in melanoma cells. Thus, the induction of ferroptosis in cells increased the effectiveness of immunotherapy. IFNγ secreted by T cells reduced SLC3A2 and SLC7A11 expression, thereby reducing the uptake of cyctine, which affects intracellular GHS levels and lipid peroxidation. Transcriptome analysis in nivolumab-treated melanoma patients showed the benefits of increasing IFNγ and decreasing SLC3A2 expression, which improved patient survival [120].

Combining a ferroptosis inducer with immunotherapy can also enhance the anti-tumor capacity. A study demonstrated that the joint treatment of erastin with an oncolytic virus (OV)-mediated cancer therapy resulted in a synergistic effect [126].

Erastin induced cytotoxicity on melanoma cells via ferroptosis but failed to generate productive and active antitumor immunity. However, co-treatment with OV and erastin improved the efficacy of OV and increased the infiltration of immune cells.

Furthermore, targeting ferroptosis-related signaling pathways can further enhance the performance of immunotherapy. Wnt/β-catenin signaling was also proven to regulate melanoma ferroptosis by increasing lipid peroxidation production [127].

ICG001 is a Wnt inhibitor that can enhance the effectiveness of anti-PD-1 immunotherapy by facilitating ferroptosis [127]. The introduction of ferroptosis improved the response to immunotherapy as well [137].

## Conclusions and future outlooks

Melanoma cancer treatment is still a crucial challenge for humans. So far, various effective treatment approaches have been explored which most focus on apoptotic cancer cell death. Meanwhile, ferroptosis has defined which is different from apoptosis in biochemistry and morphology. Due to the fact that ferroptosis has shown good anticancer efficacy since its discovery, it can unveil a novel treatment horizon for defeating apoptosis resistance in multidrug-resistant cancers.

FDA-approved drugs altretamine, SAS, sorafenib, and nanoparticles as ferroptosis inducers in cancer build high chances for treatment of resistant cancer like melanoma. Taking into consideration these positive observations, ferroptosis is promised to be a bright melanoma treatment strategy soon, either alone or in combination therapy. However, there are still many concerns that more research is needed to address them.

## Data Availability

The data presented in this study are available in this manuscript.
